# Gut microbiota drives the attenuation of dextran sulphate sodium-induced colitis by Huangqin decoction

**DOI:** 10.18632/oncotarget.16458

**Published:** 2017-03-22

**Authors:** Yang Yang, Gang Chen, Qian Yang, Juan Ye, Xueting Cai, Pamo Tsering, Xiaolan Cheng, Chunping Hu, Shuangquan Zhang, Peng Cao

**Affiliations:** ^1^ Affiliated Hospital of Integrated Traditional Chinese and Western Medicine, Nanjing University of Chinese Medicine, Nanjing 210028, Jiangsu, China; ^2^ Laboratory of Cellular and Molecular Biology, Jiangsu Province Academy of Traditional Chinese Medicine, Nanjing 210028, Jiangsu, China; ^3^ State Key Laboratory of Pharmaceutical Biotechnology, School of Life Sciences, Nanjing University, Nanjing 210097, China; ^4^ School of Pharmacy, China Pharmaceutical University, Nanjing 210009, China; ^5^ Hainan Tibetan Autonomous Prefecture Tibetan Medical Hospital, Gonghe 813099, China; ^6^ School of Life Science, Nanjing Normal University, Nanjing 210046, China

**Keywords:** Huangqin decoction, ulcerative colitis, gut microbiota, high-throughput sequencing

## Abstract

The gut microbiota, including probiotics and pathogenic microorganisms, is involved in ulcerative colitis (UC) by regulating pathogenic microorganisms and the production of intestinal mucosal antibodies. Huangqin decoction (HQD), a traditional Chinese formula chronicled in the *Shanghan lun*, has been recognized as an effective drug for UC, owing to its anti-inflammatory and anti-oxidative properties. In the present study, we investigated whether HQD ameliorates dextran sulphate sodium (DSS)-induced colitis through alteration of the gut microbiota. We found that HQD significantly inhibited colitis, alleviating the loss of body weight, disease activity index, colon shortening, tissue injury, and inflammatory cytokine changes induced by DSS treatment. Principal component analysis and principal co-ordinate analysis showed an obvious difference among the groups, with increased diversity in the DSS and DSS+HQD groups. Linear discriminant analysis effect size was used to determine differences between the groups. The relative abundance of *Lactococcus* was higher in the DSS+HQD group than in the DSS group, whereas *Desulfovibrio* and *Helicobacter* were decreased. Furthermore, the protective effect of HQD was attenuated only in antibiotic-treated mice. In conclusion, our results suggest that HQD could ameliorate DSS-induced inflammation through alteration of the gut microbiota.

## INTRODUCTION

Inflammatory bowel disease (IBD) is a group of inflammatory conditions of the colon and small intestine that includes ulcerative colitis (UC) and Crohn's disease (CD) [1]. UC mainly begins in the rectum, spreads proximally in a continuous fashion, and frequently involves the periappendiceal region. It is characterized by acute pain, vomiting, weight loss, diffuse mucosal inflammation, diarrhoea, and bloody stools [2, 3]. Accumulated studies have shown that the major UC-triggering factors are immunization, apoptosis, heredity, physical environment, and infection [4]. Significant progress has been made in understanding the pathogenesis of human IBD, but its precise aetiology remains unknown.

The gut microbiota is part of a complex network responsible for maintaining normal physiological function. Studies indicate that the gut microbiota responds to changes in the host and can produce vitamins and active substances to combat pathogens by taking energy from food [5]. Coexistence between the host and the gut microbiota helps to shape the mucosal and systemic immune systems. When the gut microbiota invades intestinal tissue and induces local or systemic inflammation, the mucosal immune system has a number of protective mechanisms that allow the host to mount an appropriate immune response to invading bacteria [6]. The intestinal microbiota is considered to be a significant factor in the aetiology of IBD [7].

There are more than 100 trillion microbes in the human gut [8]. Of these microorganisms, which include probiotics and pathogens, Bacteroidetes and Firmicutes are the predominant members; Proteobacteria is the third most common phylum. In recent years, an increasing number of gut microorganisms have been detected with high throughput sequencing. Metagenomic and 16S rRNA-based marker gene sequencing studies have demonstrated reductions in biodiversity in patients with IBD compared with healthy individuals [9]. Recent studies have demonstrated that there is a significant difference in the relative abundance of Bacteroidetes and Firmicutes in active UC compared with the control [10]. Moreover, a significant increase in *Fusobacterium* colonisation, possibly associated with T-cell response and microRNA expression, has been found in colon cancer tissues by metagenomic analysis [11]. The gut microbiota has become an important target in the treatment of irritable bowel syndrome (IBS), and therapy has focused on correcting intestinal microbial imbalance. Clinically, a low FODMAP (fermentable oligo-, di-, and monosaccharides and polyols) diet has been used to reduce symptoms in patients with IBS by regulating the gut microbiota [12]. In addition, a study revealed an existing relationship between microbial agents and gastric cancer: chronic infection with gram-negative bacteria *Helicobacter pylori*, *Streptococcus mitis*, *S. parasanguinis*, *Lactobacillus*, *Veillonella*, and *Prevotella* dominates flora in the microbiota of people who develop gastric cancer [13–15]. The results of faecal sample testing from patients with colorectal cancer (CRC) and healthy individuals have shown that *Enterococcus* and *Streptococcus* are increasingly present in samples from CRC patients, while the butyrate-producing bacteria *Roseburia* and *Clostridium* predominate in the control samples [16]. Recent metagenomic studies have demonstrated that there are differences in gut microbiota between lean and obese individuals [17]. In addition, the gut microbiota has a close relationship with type 2 diabetes. Expression levels of genes involved in carbohydrate and sulphur metabolism are increased in patients with diabetes compared with that in control individuals [18].

Huangqin decoction (HQD), a classical traditional Chinese herbal formulation, is widely used to ameliorate gastrointestinal disorders such as IBD. It has been proven that HQD is able to inhibit the expression levels of pro-inflammatory cytokines and the relative activity of NF-κB p65 in mice [19, 20]. A recent study has suggested that HQD can effectively inhibit the up-regulation of the Wnt/β-catenin signalling pathway induced by unilateral ureteral obstruction in mice, which suggests that HQD might have a role in improving renal interstitial fibrosis [21]. The active constituents in HQD, including bacalin, wogonoside, oroxylin-A, paeonimetabolin-I, liquirigenin, and glycyrrhetinic acid, have been determined using high-performance liquid chromatography (HPLC) [22]. In our previous study, HQD inhibited the development of acute/chronic colitis and prevented colitis-associated CRC, possibly by inhibiting inflammation and preventing oxidative stress-induced cellular damage [23]. The relationship between HQD and the gut microbiota in colitis was, however, not clear. In this study, we aim to investigate changes in the microbiota when dextran sulphate sodium (DSS)-induced colitis is ameliorated by HQD.

## RESULTS

### HQD ameliorates colitis and regulates cytokine expression levels in the colon of mice with DSS-induced colitis

**Figure 1:**
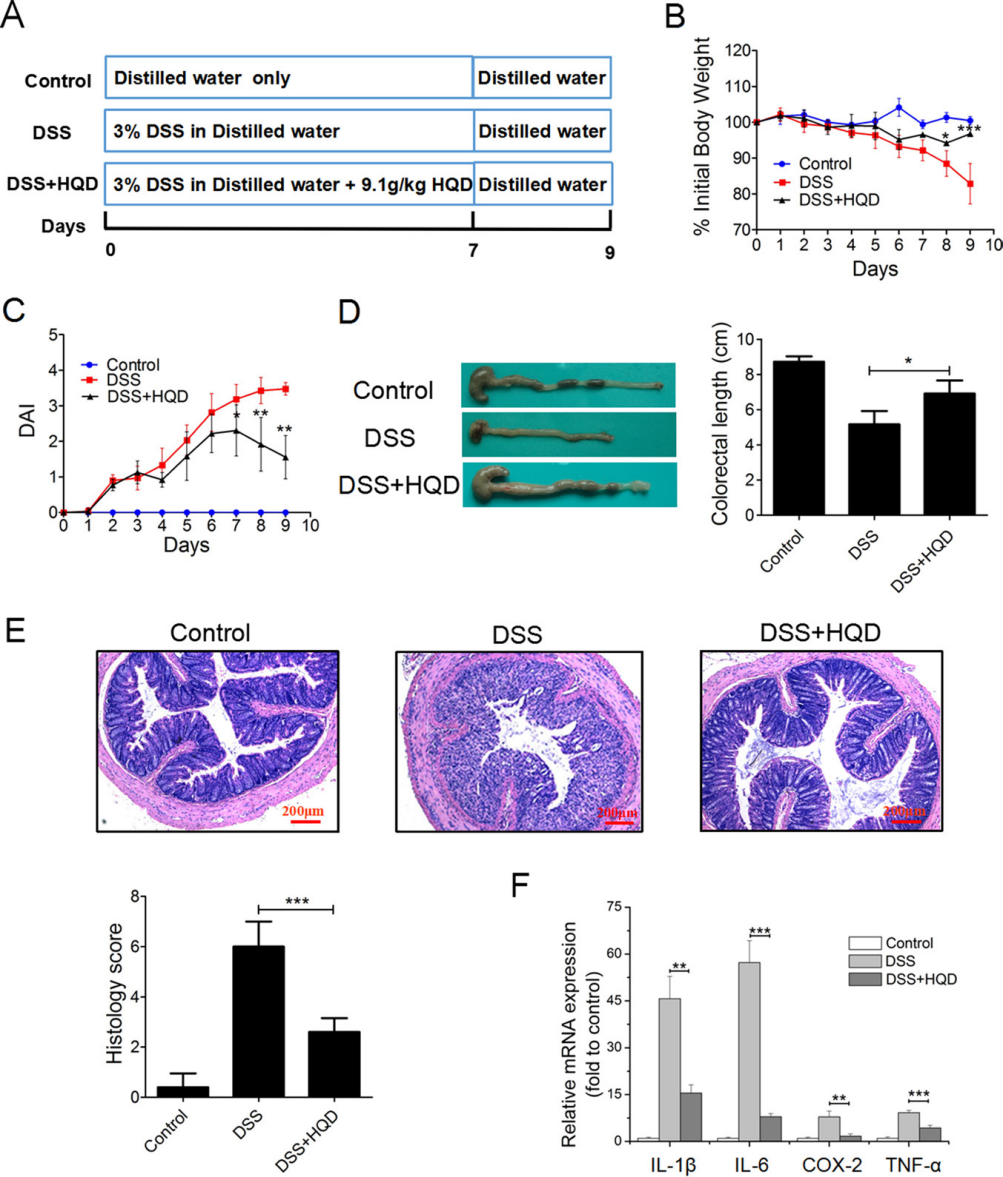
Role of HQD against DSS-induced colitis in C57BL/6 mice (**A**) Experimental process inducing UC by 3% DSS. (**B**) Body weight change after 3% DSS induction of colitis. (**C**) DAI and (**D**) intestine photograph and statistics of colorectum length in each group. (**E**) Representative H&E-stained colorectum sections (100× magnification) in mice with acute colitis. Histology score based on ten H&E-stained sections per mouse. (**F**) Expression levels of TNF-α, IL-1β, IL-6, and COX-2 in colorectum of acute colitis model determined by RT-qPCR. *P < 0.05; **P < 0.01; ***P < 0.001, versus DSS-treated group. Data are presented as mean ± SD of six mice in each group (B-F, *n* = 6). HQD, Huangqin decoction.

Colitis was induced in mice by administration of 3% DSS in drinking water for 7 days (Figure 1A). During modelling, no difference in body weight was detected between the HQD group and control group mice in the first 5 days; a significant decrease in body weight was then observed in DSS-treated mice. The mice in the HQD group experienced less weight loss compared with the DSS group mice at 7–9 days (Figure 1B). Generally, disease activity index (DAI) exhibited features of loose faeces, haematochezia and body weight reduction, and was used to evaluate inflammation severity in mice with colitis. The DAI score increased significantly after DSS intake, whereas it was markedly attenuated in the HQD-treated group (Figure 1C). Colon shortening is another index that reflects the severity of colorectal inflammation. Figure 1D shows that a significant shortening of the colon was observed in the DSS group compared with the control and HQD groups. H&E-stained colorectal sections showed that mice from the DSS group exhibited distortion of crypts, loss of goblet cells, severe epithelial injury, and inflammatory cell infiltration in the mucosa and submucosa. The HQD group exhibited obvious protection of the colon crypt structures and less severe histologic inflammation (Figure 1E). Tumour necrosis factor alpha (TNF-α), Interleukin 6 (IL-6), Interleukin 1 beta (IL-1β), and cyclooxygenase-2 (COX-2) play a pivotal role in the onset of colitis. Compared with the control group, the levels of TNF-α, IL-6, IL-1β, and COX-2 were significantly increased in the colon of the DSS group. The administration of HQD, however, significantly suppressed the accumulation of TNF-α, IL-6, IL-1β, and COX-2 in the colon tissues of mice with DSS-induced colitis. These results suggest that HQD is capable of preventing DSS-induced colitis (Figure 1F). Taken together, our data suggest that HQD exerts an anti-inflammatory action in DSS-induced colitis and is capable of preventing DSS-induced colitis.

### Overall structural modulation of gut microbiota after HQD treatment

**Figure 2:**
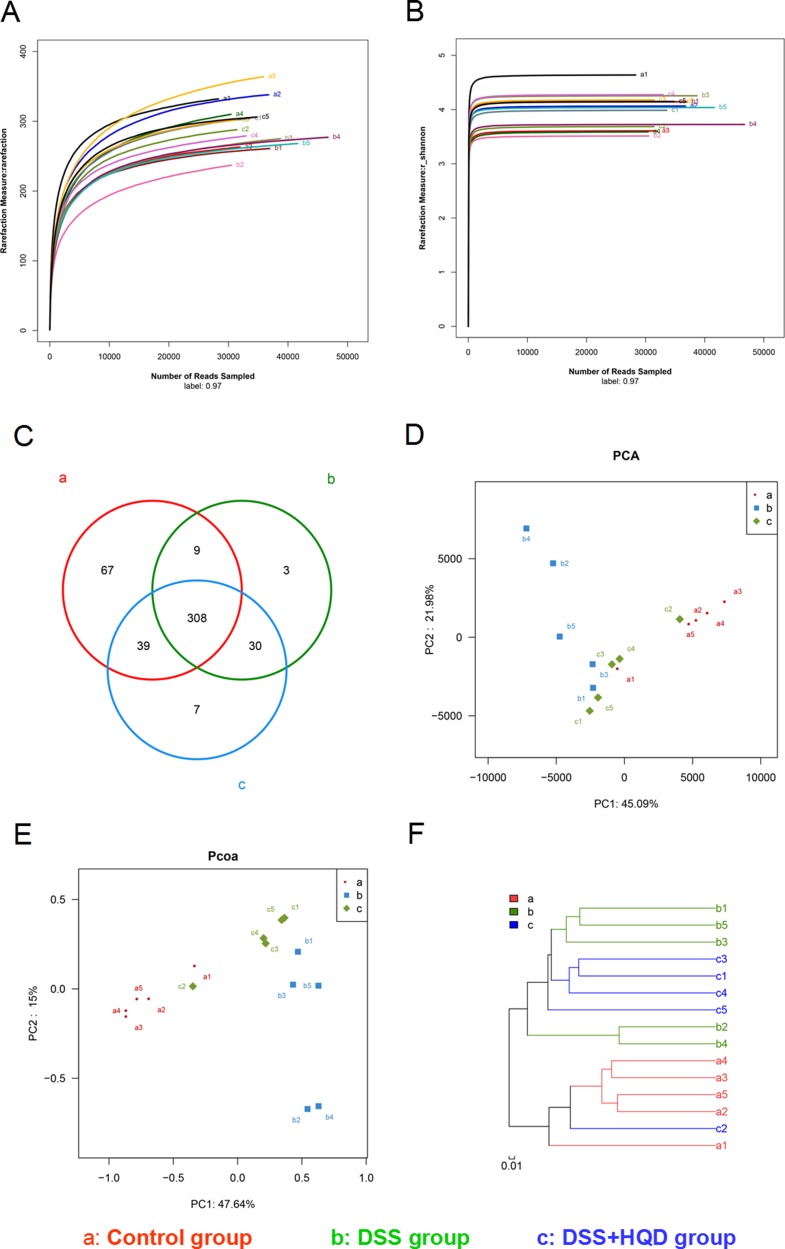
Evaluation of illumina MiSeq sequencing data showing that HQD could modulate the overall structure of gut microbiota (**A**) Rarefaction curves determined at the 97% similarity level. (**B**) Shannon–Wiener curves of samples, calculated using mothur. (**C**) Venn diagram of OTUs in the three groups. (**D**) Multiple sample PCA analysis. (**E**) Multiple sample PCoA analysis. (**F**) Multiple sample similarity tree. Samples of the control group indicated by a, samples of the DSS group by b, and samples of the DSS+HQD group by c. HQD, Huangqin decoction.

We conducted a bar-coded pyrosequencing run to analyse structural changes in the gut microbiota in the three studied groups. In total, 746,978 usable reads and 463 OTUs were obtained from the 15 samples. Rarefaction and Shannon diversity curves are shown in Figure 2A and 2B. The rarefaction curves plateau with the current sequencing, indicating that most of the diversity has already been captured in all samples. In addition, the overlap of OTUs between groups revealed that 308 OTUs coexisted in all three groups. A further 317 OTUs were present in both the control and DSS groups, 338 in the DSS and HQD groups, and 347 in the control and HQD groups (Figure 2C). PCA and PCoA analyses revealed that the gut microbiota in the DSS group deviated from the baseline structure, and the HQD group did not return to the level of the control group (Figure 2D and 2E). The system clustering tree showed that a significant difference existed in the three groups, and the level of the HQD group was close to that of the control group (Figure 2F).

### HQD regulates structural segregation of gut microbiota in mice

**Figure 3:**
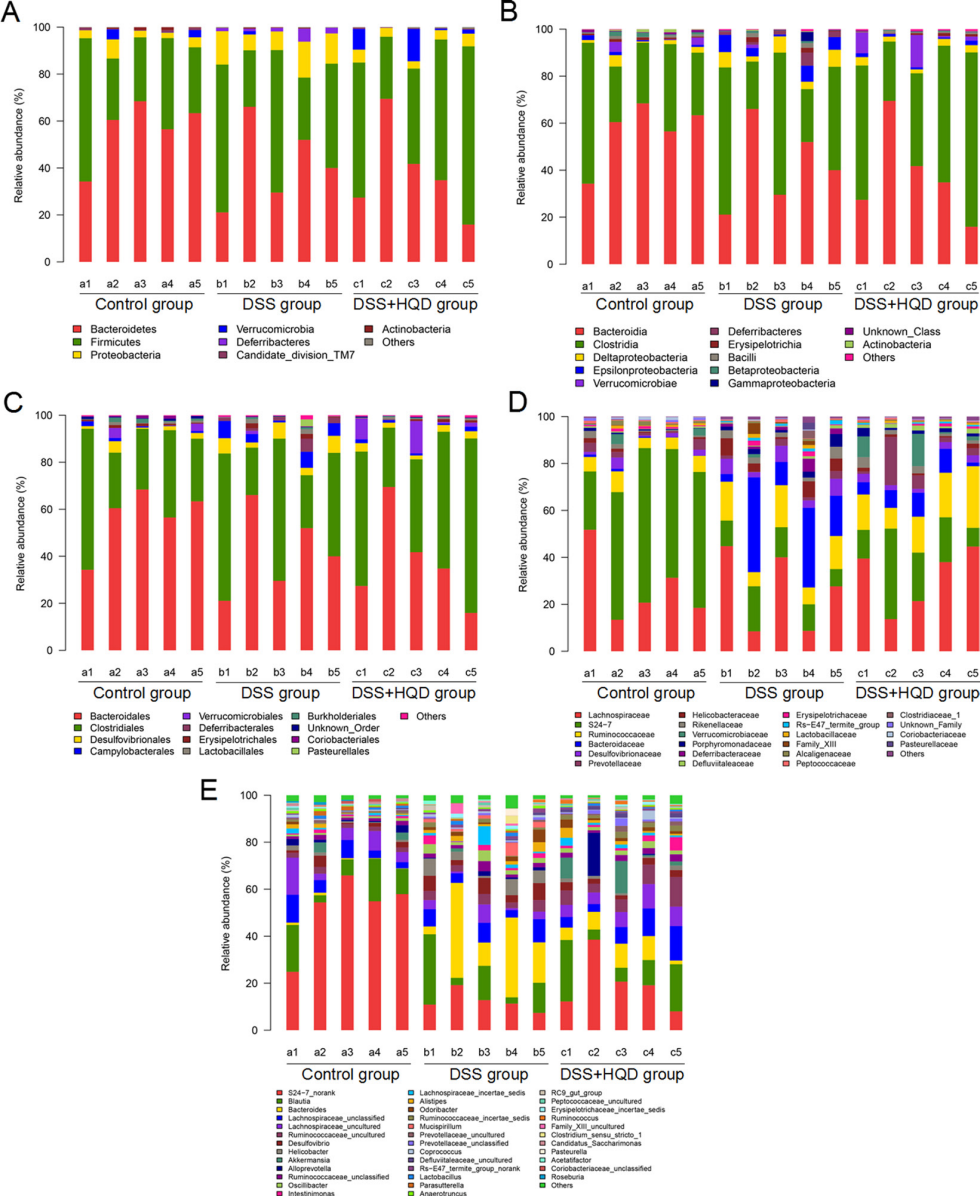
Gut microbial community structure in mice after HQD treatment (**A**) Microbial community bar plot by phylum. (**B**) Microbial community bar plot by class. (**C**) Microbial community bar plot by order. (**D**) Microbial community bar plot by family. (**E**) Microbial community bar plot by genus. Control-a, DSS-b, DSS+HQD-c. HQD, Huangqin decoction.

Histograms illustrating the gut microbiota community structure reveal the microbial species and their relative abundance. As shown in Figure 3, all samples contained Bacteroidetes, Firmicutes, Proteobacteria, Verrucomicrobia, candidate division TM7, and Actinobacteria. The most abundant phyla were Bacteroidetes, Firmicutes, and Proteobacteria. At the class level, 12 classes including Bacteroidia and Clostridia were found in all samples. Analysis of results shows that the relative abundance of Deferribacteres and Epsilonproteobacteria was significantly different among the three groups. Similarly, sequencing data identified 21 families of microbial flora. Anaeroplasmatales, Enterobacteriales, and Rhodospirillales strains were identified in the DSS and HQD groups but were not detected in the control group. Conversely, Bacteroidales and Bifidobacteriales were detected in the control group but not in the other groups. As shown in Figure 3D, Lactobacillaceae, S24-7, Ruminococcaceae, and Bacteroidaceae strains accounted for the majority of the 22 orders of microflora. Finally, 37 genera were identified in all samples. *S24-7_norank* was found at significantly lower levels in the DSS group compared with that in the control group. Conversely, the relative abundance of *Bacteroides* was significantly higher in the DSS group compared with the control and HQD groups.

### HQD inhibits the growth of certain bacteria

**Figure 4:**
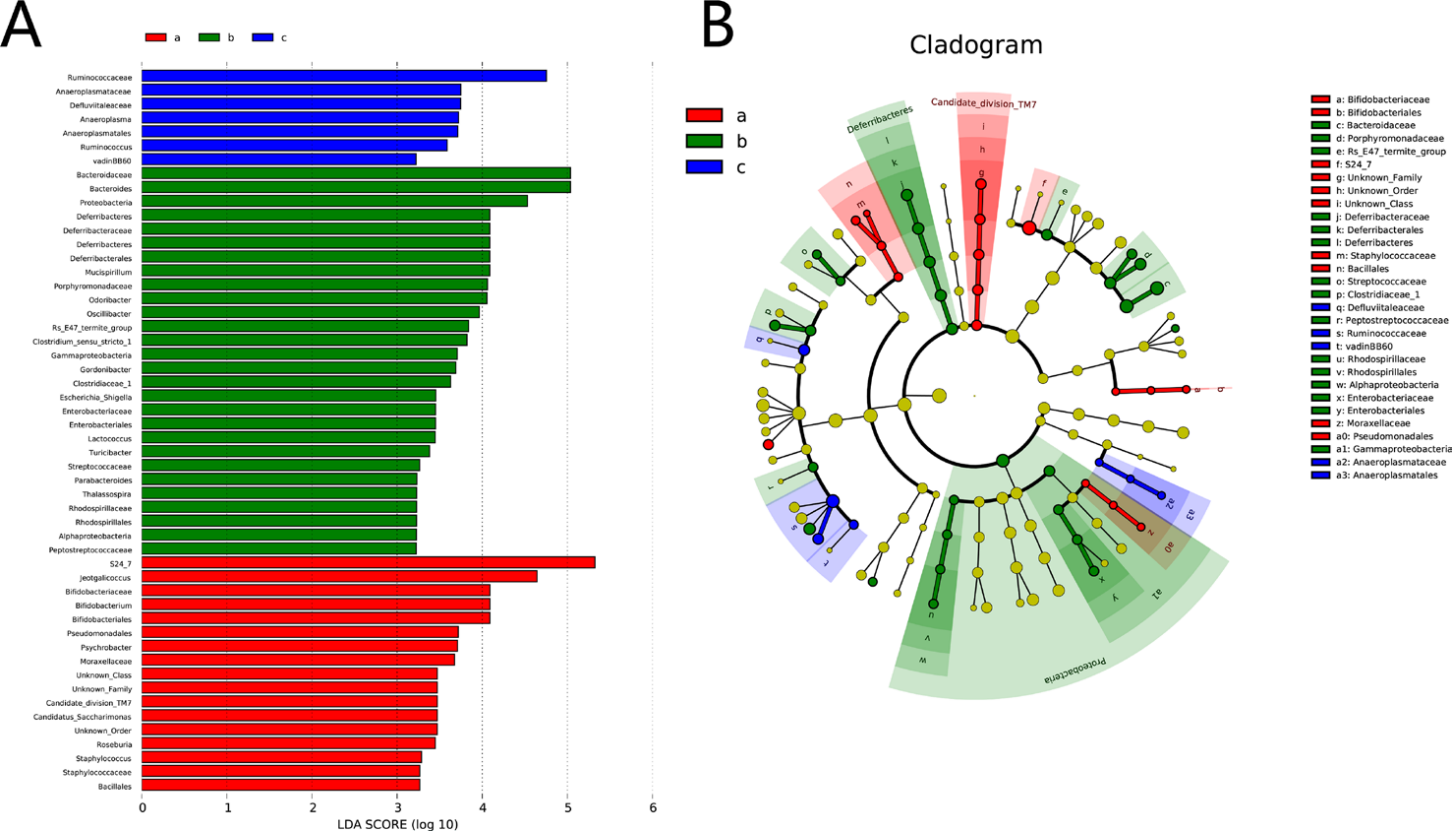
Difference in dominant microorganisms between groups (**A**) Distribution histogram based on LDA. (**B**) Cladogram. Control-a, DSS-b, DSS+HQD-c. HQD, Huangqin decoction.

**Figure 5:**
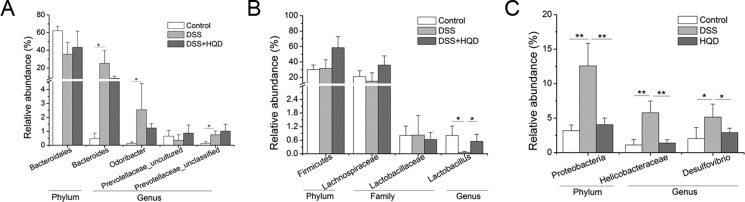
Effect of HQD on gut microbial relative abundance in mice (**A**) Relative abundance within Bacteroidetes. (**B**) Relative abundance within Firmicutes. (**C**) Relative abundance within Proteobacteria. *P < 0.05; **P < 0.01; ***P < 0.001. Data are presented as mean ± SD of five soil samples in each group (A–C, *n* = 5). HQD, Huangqin decoction.

In the experiment, the gut microbiota diversity in the groups studied was analysed by LEfSe (LDA Effect Size). A histogram of LDA scores was plotted to identify statistically significant biomarkers and to reveal the dominant microorganisms in the groups. The results show that 17 taxa were found in the control group but not the DSS and HQD groups. S24_7, *Jeotgalicoccus*, and Bifidobacteriaceae had a great influence in the dominant community. Dominant communities of 28 taxa and seven taxa were found in the DSS and HQD groups, respectively. Among them, Bacteroidaceae and *Bacteroides* had an important influence in the DSS group. Ruminococcaceae and Anaeroplasmataceae were the dominant flora in the HQD group. An evolutionary clustering analysis diagram was generated based on the LDA score to identify important microflora using taxonomy. As shown in Figure 4, the branches of candidate division TM7, Bacillales, and Pseudomonadales were the major microbiota in the control group. In the DSS group, the predominant intestinal flora, including Deferribacteres, Porphyromonadaceae, Bacteroidaceae, Enterobacteriales, and Rhodospirillales, played an important role in the active period of colitis. The branches of Anaeroplasmataceae and *Ruminococcus*, however, were identified as novel superior microbiota in the DSS+HQD group. Furthermore, the results in Figure 5A–5C show that the relative abundance of *Bacteroides*, *Odoribacter*, and *Prevotellaceae_uncultured* in the DSS group was markedly higher than in the control group, and there was no statistical difference compared with the DSS+HQD group. The situation was similar for the relative abundance of *Helicobacter* and *Desulfovibrio*, but these were significantly decreased with HQD. Taken together, these results show that HQD could change the microbial composition in the intestine and inhibit the proliferation of certain bacteria.

### Gut microbiota drives attenuation of DSS-induced ulcerative colitis by HQD

In previous experiments, it has been found that HQD ameliorates DSS-induced acute colitis in mice by inhibiting pro-inflammatory factors. Moreover, HQD could change the composition of the gut microbiota and inhibit the growth of certain bacteria. We therefore carried out further experiments to prove the relationship between the gut microbiota and the anti-inflammatory effects of HQD.

**Figure 6:**
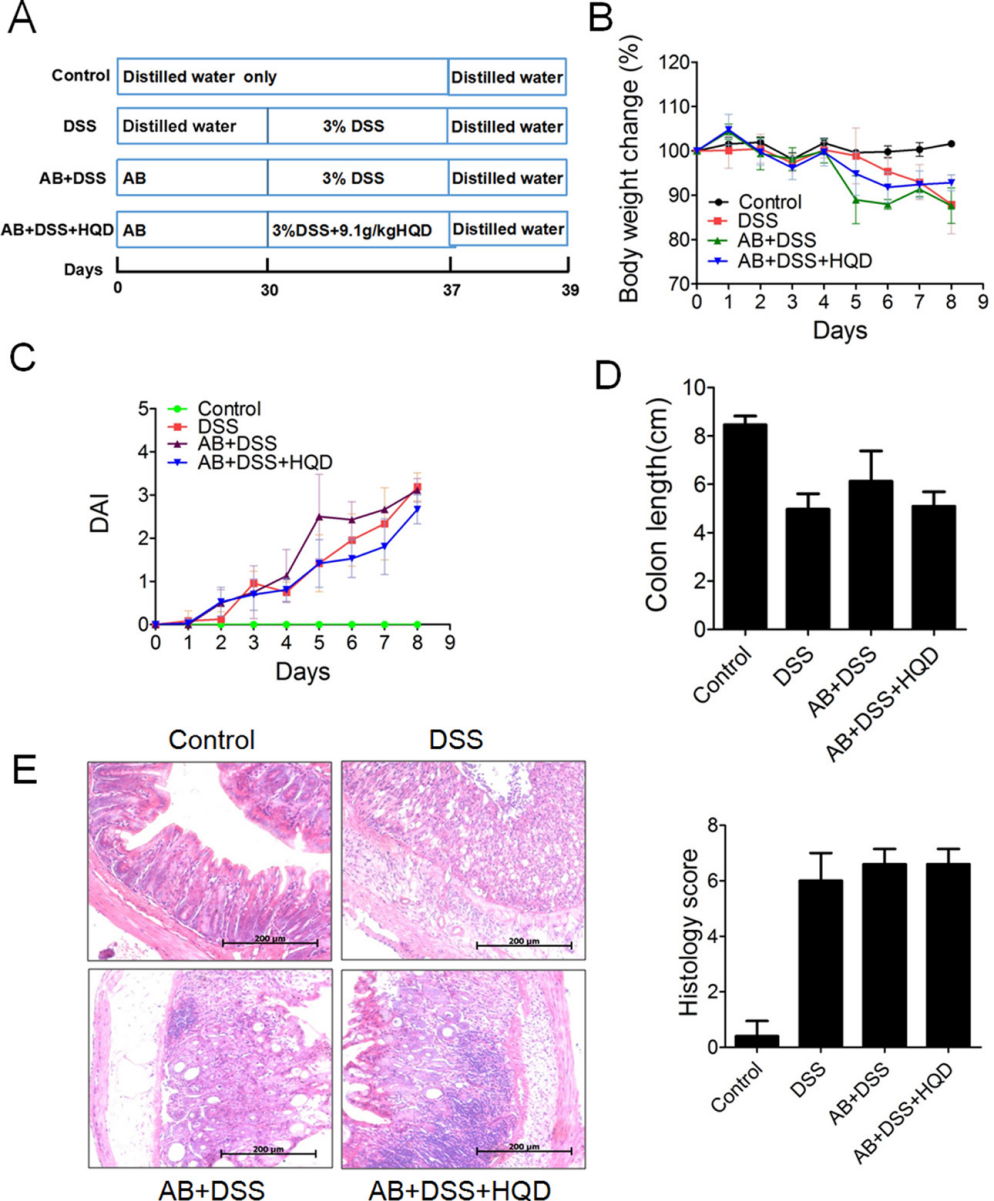
Effect of HQD against DSS-induced colitis after pretreatment with AB (**A**) Experimental process inducing colitis by 3% DSS after pretreatment with AB. (**B**) Body weight change after 3% DSS induction of colitis after AB. (**C**) DAI. (**D**) Statistics of colorectum length in each group. (**E**) Representative H&E-stained colorectum sections (100× magnification) and Histology score based on ten H&E-stained sections per mouse. *P < 0.05; **P < 0.01; ***P < 0.001. Data are presented as mean ± SD of six soil samples in each group (B-E, *n* = 6). AB, antibiotic. HQD, Huangqin decoction.

Mice were administered a broad-spectrum antibiotic (AB) in drinking water for 30 days [24]. When we cultured the faeces, almost no bacterial growth was found (Supplementary Figure 1). Colitis was then induced by administration of 3% DSS in drinking water for 7 days (Figure 6A). This resulted in a significant decrease in body weight in DSS-treated mice. At 5–6 days, the mice in the AB+DSS group exhibited significant weight loss compared with the DSS group mice (Figure 6B). Although gradual weight loss was observed in the AB+DSS+HQD group, there was no obvious difference compared with the DSS group. As shown in Figure 6C, the DAI score increased significantly after DSS intake, whereas it was markedly increased in the AB+DSS group. Figure 6D shows that a significant shortening of the colon was observed in the DSS, AB+DSS and AB+DSS+HQD groups compared with that in the control group. Similarly, H&E-stained colon sections showed that all groups exhibited distortion of the crypts, loss of goblet cells, severe epithelial injury, and inflammatory cell infiltration in the mucosa and submucosa (Figure 6E). Thus, in microbiota-depleted conditions, the sensitivity to DSS-induced colitis was increased, and HQD did not prevent DSS-induced colitis. The above results suggest that the gut microbiota drives the attenuation of DSS-induced ulcerative colitis by HQD.

## DISCUSSION

Inflammatory bowel disease, including UC and CD, is a chronic inflammatory condition of the gastrointestinal tract. In recent years, many studies have shown that UC is associated with genetic and environment factors that lead to impairment of the intestinal mucosal barrier. Previous studies have reported that IL-10^−/−^, TRAF6^IEC-KO^ and TMF^−/−^ mice are susceptible to colitis [24, 25]. Moreover, it has been confirmed that attenuation of the Bridging integrator 1 gene can limit UC pathogenicity in mice by supporting mucosal barrier function and protecting the integrity of the lymphoid follicle, thereby offering a novel strategy to treat UC and possibly limiting the risk of colorectal cancer.

Although UC is generally treated with anti-inflammatory or immunosuppressive drugs, most of these treatments often prove to be inadequate and have various side effects. Consequently, many traditional plant-based remedies have recently been explored as alternative treatments. Many studies have investigated the mechanism of action of traditional Chinese medicine on UC. Our previous studies have shown that *Banxia xiexin* decoction and Huangqin decoction could protect against DSS-induced colitis through their anti-inflammatory and anti-oxidant activities [23, 26]. It has been demonstrated that Huangqin Tang could inhibit the relative activity of NF-κB p65 and decrease the expression levels of NO, IL-6, TNF-α, and COX-2. An increasing number of studies have recently focused on the relationship between UC and the gut microflora [27]. The gut microbiota, including symbiotic, probiotic, and pathogenic microorganisms, plays an important role in human health. Its balance can be destroyed by the introduction of invasive antigens into the organism, activation of immune cells, and production of cytokines. Therefore, it is of great significance to investigate the relationship between HQD and the gut microbiota in DSS-induced colitis in mice.

In this study, we investigated the anti-inflammatory role of HQD in the *in vivo* model of colitis induced by DSS in C57BL/6 mice. After treatment with DSS, the mice exhibited clinical symptoms and pathological changes corresponding to those of human UC, including loss of body weight, diarrhoea, bloody faeces, mucosal ulceration, and shortening of the colon. Two different concentrations of HQD along with DSS were used to evaluate its therapeutic effect on UC. The results demonstrate that HQD significantly suppresses DSS-induced colitis by decreasing DAI scores, weight loss, and colonic shortening. The results of histopathology findings were consistent with DAI data. As shown in Figure 1, the colons of mice treated with DSS presented more severe inflammatory cell infiltration, mucosal erosion, and both distortion and loss of crypts. In contrast, administration of HQD reduced all of the above-mentioned inflammatory changes, suggesting that HQD could ameliorate the inflammatory condition induced by DSS. Furthermore, the expression levels of pro-inflammatory cytokines (TNF-α, COX-2, IL-6, and IL-1β) were measured in colonic tissue [26]. The results of RT-qPCR confirmed the up-regulation of pro-inflammatory cytokines in the DSS group and their down-regulation in the HQD administrated group.

Microbiological composition was determined in mice by high-throughput sequencing. As shown in Table 1, the Simpson and Shannon indices reveal that the microbial diversity in DSS and DSS+HQD groups was greater than in the control. The PCA and PcoA analyses suggest that a significant distance existed between the three groups, with a shorter distance between the control and HQD groups. The gut microbiota community in all samples was evaluated based on the following criteria: phylum, class, order, family, and genus. In accordance with previous research, Bacteroidetes, Firmicutes, and Proteobacteria were the dominant phyla found in the gut, while Verrucomicrobia, candidate division TM7 and Actinobacteria occupied a second tier^10^. Statistical results reveal that there was no difference between the groups in abundance of Bacteroidetes and Firmicutes, which is inconsistent with previous studies. We speculate that the possible cause behind this finding is a difference in feeding environment. Our results have also shown, however, that HQD could change the relative abundance of *Helicobacter*, *Desulfovibrio* and *Lactobacillus* strains. *Helicobacter* pylori is a bacterium that causes chronic gastritis and peptic ulcer disease and, to a lesser extent, mucosa-associated lymphoid tissue (MALT) lymphoma and gastric cancer in infected individuals. Numerous studies have reported that the prevalence of *H*. *pylori* infection is lower in patients with IBD compared to controls [28], whereas *Helicobacter* infection is higher in mice with colitis compared to the control and DSS+HQD groups. The reason behind the difference is that our result is based on the gut microbiota, while previous studies focused on the serum of patients. Therefore, the relationship between *Helicobacter* and colitis needs to be investigated further. *Desulfovibrio*, a type of intestinal sulphate-reducing bacteria harmful to colonic epithelial cells, was more prevalent in mice with colitis induced by DSS. As shown by our results, the relative abundance of *Desulfovibrio* increased significantly in the DSS group but decreased significantly in the HQD group. The above results suggest that HQD could inhibit the growth of harmful bacteria in the gut. Moreover, the relative abundance of *Lactobacillus* decreased in DSS-induced colitis, which is in accordance with previous studies [29]. A significant increase, however, was found in the HQD group compared with that in the DSS group. Therefore, we suspect that HQD could promote the growth of beneficial bacteria in the gut. In a CPT-11 treated anti-colon cancer tumor allografts model, HQD changed the profile of intestinal bacterial species: *Lactobacillus*/*Enterococcus*, *Bacteroides*, *Clostridium leptum*, and *E. rectale*/*C. coccoides* [30]. A recent finding indicated that the density of *C. leptum* is significantly reduced in the faecal microbiota of patients with Crohn's disease and ulcerative colitis [31]. HQD can increase the density of *C. leptum* in colonic tissue. HQD is also able to maintain *C. leptum* levels in the colon following CPT-11 treatment. Therefore, HQD may have potential benefit in treating IBD by restoring the density of *C. leptum* in the colon [30].

**Table 1:** Diversity indices of the various groups

	OTU	Ace	Chao	Simpson	Shannon	Coverage
Control	423	430 (426,443)	432 (426,453)	0.0492 (0.0484,0.0499)	4.37 (4.37,4.38)	0.999908
DSS	350	357 (352,370)	357 (352,374)	0.0269 (0.0267,0.0272)	4.42 (4.41,4.42)	0.999933
DSS+HQD	384	393 (387,408)	395 (387,419)	0.02 (0.0198,0.0202)	4.58 (4.57,4.59)	0.999909

Several studies have reported that individual herbs of HQD possess antibiotic activity. Different chemicals derived from *Paeonia lactiflora* were found to have different activities against intestinal flora [32]. It was found that human intestinal bacteria could transform flavonoids of *Scutellaria baicalensis* into aglycone flavonoids, baicalein, oroxylin A, wogonin and norwogonin, each of which has different potencies against different types of intestinal bacteria [33]. Glycyrrhizol A and 6,8-diisoprenyl-5,7,4′-trihydroxyisoflavone (5) isolated form *Glycyrrhiza uralensis* were shown to exhibit potent antibacterial activity against *Streptococcus* mutants [34]. Crude ethanol extracts of *Ziziphus jujuba* fruits were also reported to exhibit antibacterial activity [35]. Future investigations, such as a comparison of the impacts of different herbal combinations of HQD on intestinal bacterial profiles, could help us to address the herb(s) or chemical(s) responsible for the specific antibiotic activities of HQD.

Although the effect of HQD on DSS-induced colitis and the gut microbiota was clearly revealed, the relationship between the anti-inflammatory role of HQD and the gut microbiota remained undefined. Consequently, an experiment was conducted to test the effect of HQD on DSS mice treated preliminarily with a broad-spectrum antibiotic. In our study, mice with DSS-induced colitis showed no significant distinction compared with mice from the HQD treated group, and only slight symptoms compared with the AB+DSS group. The results show no effect of HQD in mice subjected to an antibiotic and DSS. Based on the findings, we confirm that HQD exhibits anti-inflammatory *in vivo* effects by changing the gut microbiota, which might have potential applications in the treatment of UC. In future studies, we plan to apply faecal transplantation assays to the study of different herbal combinations of HQD on intestinal bacterial profiles; this could help us to clarify the detailed mechanism underlying the gut microbiota regulation activities of HQD.

## MATERIALS AND METHODS

### Chemicals and reagents

DSS (MW 36,000–50,000) was obtained from Sigma-Aldrich, USA. E.Z.N.A.^®^ Soil DNA Kit was purchased from OMEGA, USA. Maxima^®^ SYBR Green/ROX qPCR Master Mix (2×) and Maxima^®^ First Strand cDNA Synthesis Kit were purchased from Fermentas Life Science (Waltham, MA, USA). The AxyPrep DNA Gel Extraction Kit was purchased from Axygen Biosciences, Union City, CA, USA. Ampicillin and neomycin were purchased from Sangon Biotech (Shanghai) Co., Ltd. Meronem and vancomycin were provided by Affiliated Hospital of Integrated Traditional Chinese and Western Medicine, Nanjing University of Chinese Medicine (Nanjing, Jiangsu, China).

### Preparation of HQD

All four medical plants, namely *Scutellaria baicalensis* Georgi, *Paeonia lactiflora* Pall, *Glycyrrhiza uralensis* Fisch, and *Ziziphus jujuba* Mill were provided by the Jiangsu Province Academy of Traditional Chinese Medicine (Nanjing, Jiangsu, China); the detailed composition of HQD is listed in Supplementary Table S1. The HQD was soaked in ten-fold distilled water for 30 min before heating. The herbs were then heated to 100°C for 30 min; the decoction was filtered through a multi-layer gauze. The residue was diluted eight-fold using distilled water for a second extraction. The filtrates were obtained under the same conditions and those obtained from two cycles of extraction were mixed for use.

Waters e2695 Alliance HPLC system (Waters Corp., MA, USA) with a 2489 UV/Vis DAD detector was used for qualitative analysis of HQD aqueous extracts. Extracts were separated by an Inertsil ODS-SP C18 column (250 mm × 4.6 mm, 5μm). The injection volume was 10 μl. The mobile phase consisted of linear gradients of 0.1% (v/v) formic acid (A) and acetonitrile (B): 0–15 min, 100–95% A (v/v), 0–5% B (v/v); 15–30 min, 95–85% A, 5–15% B; 30–60 min, 85–77% A, 15–23% B; 60–90 min, 77–55% A, 23–45% B; 90–110 min, 65–40% A, 45–60% B; 110–115 min, 40–90% A, 60–5% B; 115–120 min, 40–90% A, 60–5% B. The mobile phase flow rate was 1 mL/min. The column was run at 30°C. Single herbal, HQD extracts, and purified chemical reference substances (Supplementary Figures 2, 3 and Supplementary Table 2) were used for quantitation by HPLC. The validation of this quantitative HPLC method showed that the method was sensitive, precise, and stable (Supplementary Table 3) [23, 36, 37].

### Animal model of colitis and treatment

All procedures involving animals were approved by the Institutional Animal Care and Use Committee of the Jiangsu Province Institute of Traditional Chinese Medicine and written up following the ARRIVE guidelines. Experiments were performed in accordance with published National Institutes of Health guidelines. 6–8-week-old C57BL/6 male healthy mice were used to adapt to the laboratory conditions for 1 week prior to the experiments. Ulcerative colitis was induced in mice by administering 3% DSS (W/V) solution in distilled water for 7 days. In the HQD group, the mice received 9.1 g/kg HQD via oral gavage daily along with DSS. Mice in control group and DSS group consumed the same volume of water as controls. In the last two days, mice in the experiments were given distilled water (Figure 1A).

For antibiotic treatment, 1 g/L ampicillin (ICN Biomedicals), 1 g/L neomycin (Sigma), 0.5 g/L meronem (AstraZeneca) and 0.5 g/L vancomycin (Sigma) were added to the drinking water for 30 days. AB-containing drinking water was refreshed every second day [24].

### Evaluation of DSS colitis

During all experiments, daily routine clinical evaluations were performed, including body weight measurement, stool characteristics and hematochezia determination. The evaluation of disease activity index (DAI) was performed using Murano's methods by combining the scores of weight loss, stool consistency and rectal bleeding. Animals were sacrificed by cervical dislocation under anaesthesia. The colon was removed and its length was determined. Luminal contents were removed from the cecum and colon, placed in sterile tube and stored at −80°C. The distal colon was fixed in 4% paraformaldehyde overnight and embedded in paraffin for routine H&E histopathologic examination to arrive at a histologic score as follows. For cell infiltration of inflammatory cells, rare inflammatory cells in the lamina propria were counted as 0; increased numbers of inflammatory cells, including neutrophils in the lamina propria as 1; confluence of inflammatory cells, extending into the submucosa as 2; and a score of 3 was given for transmural extension of the inflammatory cell infiltrate. For epithelial damage, absence of mucosal damage was counted as 0, discrete focal lymphoepithelial lesions were counted as 1, mucosal erosion/ulceration was counted as 2, and a score of 3 was given for extensive mucosal damage and extension through deeper structures of the bowel wall. The two subscores were added and the combined histologic score ranged from 0 (no changes) to 6 (extensive cell infiltration and tissue damage) [38]. The expression levels of inflammatory cytokines, including IL-1β, IL-6, COX-2 and TNF-a, were detected by Real-time qPCR. Primers used for the reactions were purchased from Genscript and the primer sequences are listed as followed: IL-1β(Forward: 5′- ACTCATTGTGGCTGTGGAGA-3′, Reverse: 5′- TTGTTCATCTCGGAGCCTGT -3′), IL-6 (Forward: 5′- CTGCAAGAGACTTCCATCCAGTT-3′, Reverse: 5′- GAAGTAGGGAAGGCCGTGG-3′), COX-2(Forward: 5′- CACCCTGACATAGACAGTGAAAG-3′, Reverse: 5′- CTGGGTCACGTTGGATGAGG-3′), TNF-a(Forward: 5′- GACCCCTTTACTCTGACCCC-3′, Reverse: 5′- AGGCTCCAGTGAATTCGGAA -3′), β-actin(Forward: 5′- TCCTGTGGCATCCACGAAACT -3′, Reverse: 5′- GAAGCATTTGCGGTGGACGAT -3′). ALL genes' mRNA expression was normalized against β-actin expression.

### Faecal DNA extraction and Illumina Miseq sequencing

Genomic DNA was extracted from every stool sample using the E.Z.N.A.^®^ Soil DNA Kit according to manufacturer's protocols (Omega Bio-tek, Norcross, GA, USA). The primers 338F (5′-barcode- ACTCCTACGGGAGGCAGCA-3′) and 806R (5′-GGACTACHVGGGTWTCTAAT -3′), where barcode is an eight-base sequence unique to each sample, were used to amplify the V3-V4 region of the bacteria 16S ribosomal RNA gene. The steps of PCR as follows: 95°C for 3 min, 27 cycles at 95°C for 30 s, 55°C for 30 s, and 72°C for 45 s and a final extension at 72°C for 10 min. PCR reactions were performed in triplicate: 20 μL mixture containing 4 μL of 5 × FastPfu Buffer, 2 μL of 2.5 mM dNTPs, 0.8 μL of each primer (5 μM), 0.4 μL of FastPfu Polymerase (Transgene, Beijing, China), and 10 ng of template DNA. Amplicons were extracted from 2% agarose gels and purified using the AxyPrep DNA Gel Extraction Kit (Axygen Biosciences, Union City, CA, U.S.) according to the manufacturer's instructions and quantified using QuantiFluor^™^ -ST (Promega, Wisconsin, USA). Purified amplicons were pooled in equimolar and paired-end sequenced (2 × 250) on an Illumina MiSeq platform according to the standard protocols. The raw reads were deposited into the NCBI Sequence Read Archive (SRA) database. Raw fastq files were demultiplexed, quality-filtered using QIIME (version 1.17). Reads that could not be assembled were discarded.

### Bioinformatics analysis

Operational Units (OTUs) were clustered with 97% similarity cutoff using UPARSE (version 7.1http://drive5.com/uparse/), and chimeric sequences were identified and removed using UCHIME. Phylogenetic affiliation of each 16S rRNA gene sequence was analysed by RDP Classifier (http://rdp.cme.msu.edu/) against the silva (SSU117/119)16S rRNA database using confidence threshold of 70%. The representative sequences of operational taxonomy units (OTUs) and their relative abundance were used to calculate the rarefaction analysis and Shannon diversity index by QIIME. The veen, phylogenetic tree, Principal Component Analysis (PCA) and Principal Coordinate Analysis (PCoA) were then used to analyse the diversity between groups. The dominant bacterial community difference between groups was detected using LDA Effect Size.

### Statistical analysis

Statistical analysis was performed using SPSS software version 15.0. Data were presented as mean ± SD. Unpaired Student *t* tests were used to compare the means of two groups. One-way analysis of variance and Adonis were used to compare the means of three groups. A level of *P* < 0.05 was considered as statistically significant.

## SUPPLEMENTARY MATERIALS FIGURES AND TABLES



## Abbreviations

DSS: dextran sulphate sodium
AOM: azoxymethane
HQD: Huangqin decoction
HPLC: high-performance liquid chromatography
CRC: colorectal cancer
IBD: inflammatory bowel disease
UC: ulcerative colitis
DAI: disease activity index
H&E: haematoxylin & eosin
PAS: periodic acid-Schiff stain
OTUs: Operational Units
PCoA: Principal Coordinate Analysis
PCA: Principal Component Analysis
SRA: Sequence Read Archive
TNF-α: Tumour necrosis factor alpha
IL-6: Interleukin 6
IL-1β: Interleukin 1 beta
COX-2: cyclooxygenase-2

## References

[1] Trallori G, Palli D, Saieva C, Bardazzi G, Bonanomi AG, d'Albasio G, Galli M, Vannozzi G, Milla M, Tarantino O, Renai F, Messori A, Amorosi A (1996). A population-based study of inflammatory bowel disease in Florence over 15 years (1978–92). Scand J Gastroentero.

[2] Grinspan A, Kornbluth A (2015). Positioning Therapy for Ulcerative Colitis. Curr Gastroenterol Rep.

[3] Khor B, Gardet A, Xavier RJ (2011). Genetics and pathogenesis of inflammatory bowel disease. Nature.

[4] Legaki E, Gazouli M (2016). Influence of environmental factors in the development of inflammatory bowel diseases. World J Gastrointest Pharmacol Ther.

[5] Yang AL, Kashyap PC (2015). A clinical primer of the role of gut microbiome in health and disease. Trop Gastroenterol.

[6] Reinoso Webb C, Koboziev I, Furr KL, Grisham MB (2016). Protective and pro-inflammatory roles of intestinal bacteria. Pathophysiology.

[7] Ohkusa T, Koido S (2015). Intestinal microbiota and ulcerative colitis. J Infect Chemother.

[8] Frank DN, St Amand AL, Feldman RA, Boedeker EC, Harpaz N, Pace NR (2007). Molecular-phylogenetic characterization of microbial community imbalances in human inflammatory bowel diseases. Proc Natl Acad Sci USA.

[9] Manichanh C, Rigottier-Gois L, Bonnaud E, Gloux K, Pelletier E, Frangeul L, Nalin R, Jarrin C, Chardon P, Marteau P, Roca J, Dore J (2006). Reduced diversity of faecal microbiota in Crohn's disease revealed by a metagenomic approach. Gut.

[10] Schwab C, Berry D, Rauch I, Rennisch I, Ramesmayer J, Hainzl E, Heider S, Decker T, Kenner L, Muller M, Strobl B, Wagner M, Schleper C (2014). Longitudinal study of murine microbiota activity and interactions with the host during acute inflammation and recovery. The ISME journal.

[11] Nosho K, Sukawa Y, Adachi Y, Ito M, Mitsuhashi K, Kurihara H, Kanno S, Yamamoto I, Ishigami K, Igarashi H, Maruyama R, Imai K, Yamamoto H (2016). Association of Fusobacterium nucleatum with immunity and molecular alterations in colorectal cancer. World J Gastroenterol.

[12] Halmos EP, Power VA, Shepherd SJ, Gibson PR, Muir JG (2014). A diet low in FODMAPs reduces symptoms of irritable bowel syndrome. Gastroenterology.

[13] de Martel C, Ferlay J, Franceschi S, Vignat J, Bray F, Forman D, Plummer M (2012). Global burden of cancers attributable to infections in 2008: a review and synthetic analysis. Lancet Oncol.

[14] Maldonado-Contreras A, Goldfarb KC, Godoy-Vitorino F, Karaoz U, Contreras M, Blaser MJ, Brodie EL, Dominguez-Bello MG (2011). Structure of the human gastric bacterial community in relation to Helicobacter pylori status. The ISME journal.

[15] Yang I, Nell S, Suerbaum S (2013). Survival in hostile territory: the microbiota of the stomach. FEMS microbiology reviews.

[16] Wu S, Rhee KJ, Albesiano E, Rabizadeh S, Wu X, Yen HR, Huso DL, Brancati FL, Wick E, McAllister F, Housseau F, Pardoll DM, Sears CL (2009). A human colonic commensal promotes colon tumorigenesis via activation of T helper type 17 T cell responses. Nat Med.

[17] Greenblum S, Turnbaugh PJ, Borenstein E (2012). Metagenomic systems biology of the human gut microbiome reveals topological shifts associated with obesity and inflammatory bowel disease. Proc Natl Acad Sci USA.

[18] Karlsson FH, Tremaroli V, Nookaew I, Bergstrom G, Behre CJ, Fagerberg B, Nielsen J, Backhed F (2013). Gut metagenome in European women with normal, impaired and diabetic glucose control. Nature.

[19] Chen P, Zhou X, Zhang L, Shan M, Bao B, Cao Y, Kang A, Ding A (2015). Anti-inflammatory effects of Huangqin tang extract in mice on ulcerative colitis. J Ethnopharmacol.

[20] Zou Y, Li WY, Wan Z, Zhao B, He ZW, Wu ZG, Huang GL, Wang J, Li BB, Lu YJ, Ding CC, Chi HG, Zheng XB (2015). Huangqin-Tang Ameliorates TNBS-Induced Colitis by Regulating Effector and Regulatory CD4(+) T Cells. Biomed Res Int.

[21] Jiang MQ, Wang L, Cao AL, Zhao J, Chen X, Wang YM, Wang H, Peng W (2015). HuangQi Decoction Improves Renal Tubulointerstitial Fibrosis in Mice by Inhibiting the Up-Regulation of Wnt/beta-Catenin Signaling Pathway. Cell Physiol Biochem.

[22] Zuo F, Zhou ZM, Yan MZ, Liu ML, Xiong YL, Zhang Q, Song HY, Ye WH (2002). Metabolism of constituents in Huangqin-Tang, a prescription in traditional Chinese medicine, by human intestinal flora. Biol Pharm Bull.

[23] Chen G, Yang Y, Hu C, Cheng X, Xu Y, Cai X, Wang M, Yang CS, Cao P (2016). Protective effects of Huangqin Decoction against ulcerative colitis and associated cancer in mice. Oncotarget.

[24] Vlantis K, Polykratis A, Welz PS, van Loo G, Pasparakis M, Wullaert A (2016). TLR-independent anti-inflammatory function of intestinal epithelial TRAF6 signalling prevents DSS-induced colitis in mice. Gut.

[25] Bel S, Elkis Y, Elifantz H, Koren O, Ben-Hamo R, Lerer-Goldshtein T, Rahimi R, Ben Horin S, Nyska A, Shpungin S, Nir U (2014). Reprogrammed and transmissible intestinal microbiota confer diminished susceptibility to induced colitis in TMF−/− mice. Proc Natl Acad Sci USA.

[26] Chen G, Yang Y, Liu M, Teng Z, Ye J, Xu Y, Cai X, Cheng X, Yang J, Hu C, Wang M, Cao P (2015). Banxia xiexin decoction protects against dextran sulfate sodium-induced chronic ulcerative colitis in mice. J Ethnopharmacol.

[27] Forbes JD, Van Domselaar G, Bernstein CN (2016). Microbiome Survey of the Inflamed and Noninflamed Gut at Different Compartments Within the Gastrointestinal Tract of Inflammatory Bowel Disease Patients. Inflamm Bowel Dis.

[28] Rokkas T, Gisbert JP, Niv Y, O'Morain C (2015). The association between Helicobacter pylori infection and inflammatory bowel disease based on meta-analysis. United Eur Gastroent.

[29] Hakansson A, Tormo-Badia N, Baridi A, Xu J, Molin G, Hagslatt ML, Karlsson C, Jeppsson B, Cilio CM, Ahrne S (2015). Immunological alteration and changes of gut microbiota after dextran sulfate sodium (DSS) administration in mice. Clin Exp Med.

[30] Lam W, Jiang Z, Guan F, Hu R, Liu SH, Chu E, Cheng YC (2014). The number of intestinal bacteria is not critical for the enhancement of antitumor activity and reduction of intestinal toxicity of irinotecan by the Chinese herbal medicine PHY906 (KD018). BMC Complement Altern Med.

[31] Kabeerdoss J, Sankaran V, Pugazhendhi S, Ramakrishna BS (2013). Clostridium leptum group bacteria abundance and diversity in the fecal microbiota of patients with inflammatory bowel disease: a case-control study in India. BMC Gastroenterol.

[32] Ngan LT, Moon JK, Kim JH, Shibamoto T, Ahn YJ (2012). Growth-inhibiting effects of Paeonia lactiflora root steam distillate constituents and structurally related compounds on human intestinal bacteria. World J Microbiol Biotechnol.

[33] Xing S, Wang M, Peng Y, Chen D, Li X (2014). Simulated gastrointestinal tract metabolism and pharmacological activities of water extract of Scutellaria baicalensis roots. J Ethnopharmacol.

[34] He J, Chen L, Heber D, Shi W, Lu QY (2006). Antibacterial compounds from Glycyrrhiza uralensis. J Nat Prod.

[35] Daneshmand F, Zare-Zardini H, Tolueinia B, Hasani Z, Ghanbari T (2013). Crude Extract from Ziziphus Jujuba Fruits, a Weapon against Pediatric Infectious Disease. Iran J Ped Hematol Oncol.

[36] Ye M, Liu SH, Jiang Z, Lee Y, Tilton R, Cheng YC (2007). Liquid chromatography/mass spectrometry analysis of PHY906, a Chinese medicine formulation for cancer therapy. Rapid Commun Mass Spectrom.

[37] Tilton R, Paiva AA, Guan JQ, Marathe R, Jiang Z, van Eyndhoven W, Bjoraker J, Prusoff Z, Wang H, Liu SH, Cheng YC (2010). A comprehensive platform for quality control of botanical drugs (PhytomicsQC): a case study of Huangqin Tang (HQT) and PHY906. Chin Med.

[38] Wallace BD, Wang H, Lane KT, Scott JE, Orans J, Koo JS, Venkatesh M, Jobin C, Yeh LA, Mani S, Redinbo MR (2010). Alleviating cancer drug toxicity by inhibiting a bacterial enzyme. Science.

