# Supplementary material for: Gut microbiota drives the attenuation of dextran sulphate sodium-induced colitis by Huangqin decoction
Source: Oncotarget. 2017 Mar 22;8(30):48863–74. doi: 10.18632/oncotarget.16458 (PMC5564731; doi:10.18632/oncotarget.16458)
Supplement: Supplementary file 1 [file oncotarget-08-48863-s001.pdf]

Gut microbiota drives the attenuation of dextran sulphate sodium-induced colitis by Huangqin decoction

Supplementary Materials

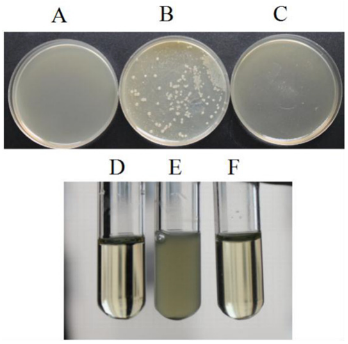

Supplementary Figure 1: Effects of antibiotic on gut bacterial growth in mice. (**A**) Sterilized water (A and **D**), faeces dilution from normal mice (**B** and **E**) and faeces dilution from antibiotic treated mice (**C** and **F**) growth in solid medium and liquid medium.

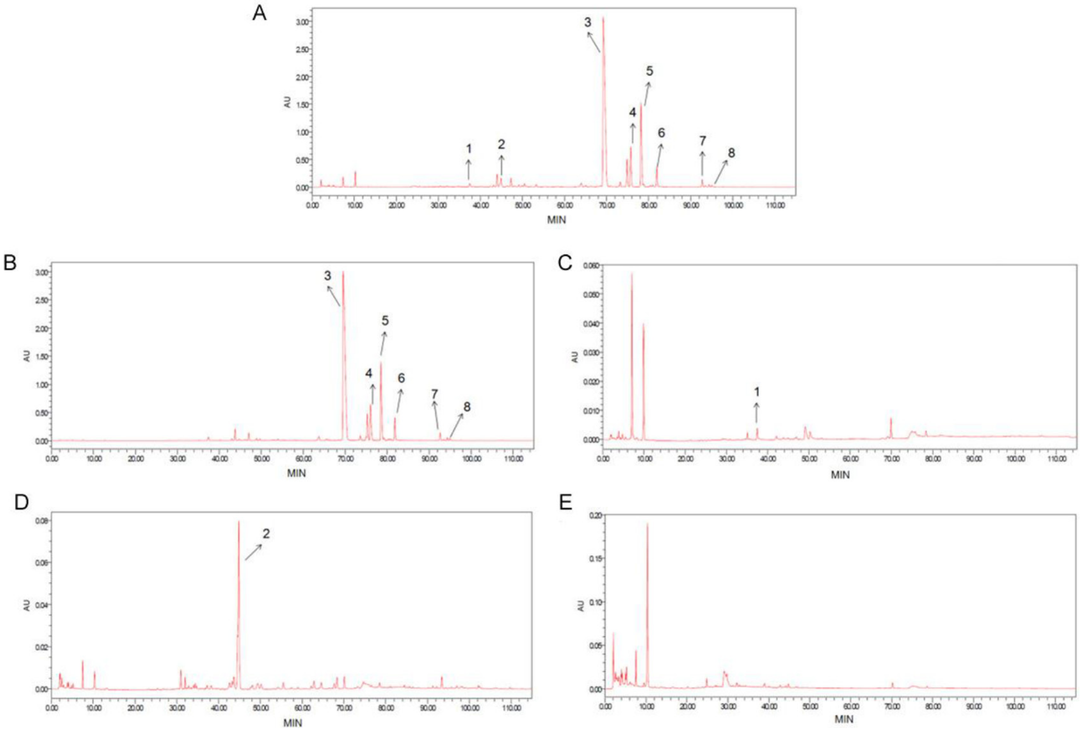

Supplementary Figure 2: HPLC chromatogram of single herbal and HQD extracts. (**A**) HQD, (**B**) *Scutellaria baicalensis Georgi*, (**C**) *Paeonia lactiflora* Pall, (**D**) *Glycyrrhiza uralensis* Fisch, (**E**) *Ziziphus jujuba* Mill.

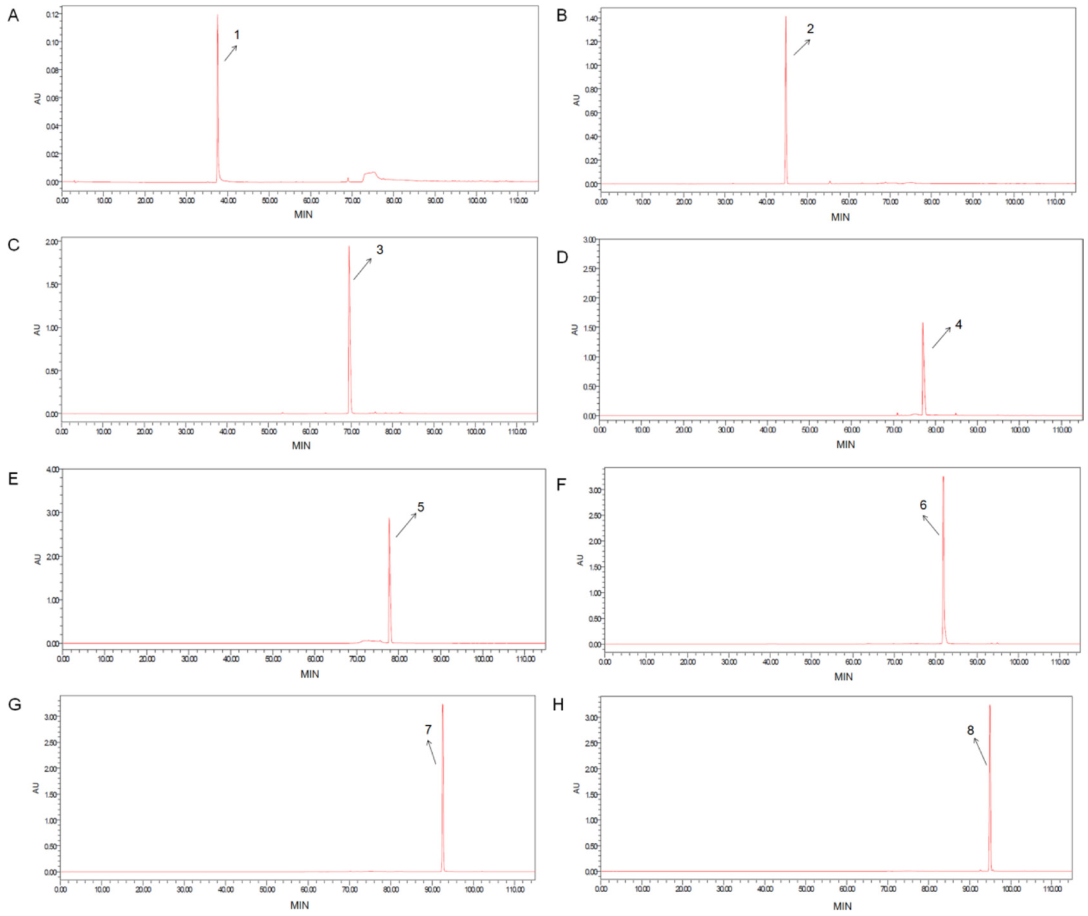

Supplementary Figure 3: HPLC chromatogram of standard. (**A**) Paeoniflorin, (**B**) Liquiritin, (**C**) Baicalin, (**D**) Oroxylin A-7-glucuronide, (**E**) Wogonoside, (**F**) Baicalein, (**G**) Wogonin, (**H**) Oroxylin A.

Supplementary Table 1: The composition of HQD

Species	Chinese name	Plant part	Grams, g
Scutellaria baicalensis Georgi	Huang qin	Root	9
Paeonia lactiflora Pall	Shao yao	Root	6
Glycyrrhiza uralensis Fisch	Gan cao	Root and rhizomes	6
Ziziphus jujuba Mill	Da zao	Fruit	49
Total amount			70

Supplementary Table 2: Compounds in HQD

No.	t_R (min)	Identification	Chemical structure	Molecular formula	Source
1	37.561	Paeoniflorin	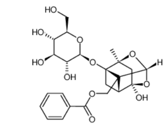	$C_{23}H_{28}O_{11}$	*Paeonia lactiflora* Pall
2	44.797	Liquiritin	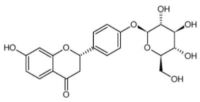	$C_{21}H_{22}O_9$	*Glycyrrhiza uralensis* Fisch
3	54.002	Baicalin	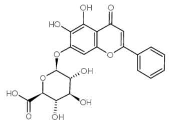	$C_{21}H_{18}O_{11}$	*Scutellaria baicalensis* Georgi
4	75.484	Oroxylin A-7-glucuronide	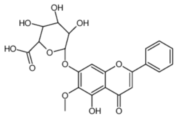	$C_{22}H_{20}O_{11}$	*Scutellaria baicalensis* Georgi
5	78.004	Wogonoside	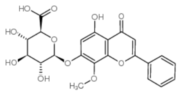	$C_{22}H_{20}O_{11}$	*Scutellaria baicalensis* Georgi
6	81.748	Baicalein	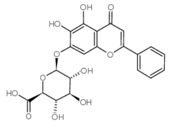	$C_{15}H_{10}O_5$	*Scutellaria baicalensis* Georgi
7	92.537	Wogonin	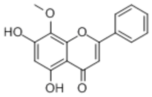	$C_{16}H_{12}O_5$	*Scutellaria baicalensis* Georgi
8	94.824	Oroxylin A	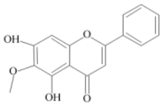	$C_{16}H_{12}O_5$	*Scutellaria baicalensis* Georgi

Supplementary Table 3: Validation of linearity, sensitivity, precision, stability and repeatability

Analyte	Linearity			Sensitivity(ng/mL)		Precision (RSD, %)		Stability (RSD,%, *n* = 6)	Repeatability (RSD,%, *n* = 3)
	Range (μg/mL)	Equation	R^2	LOD	LOQ	Intra-day (*n* = 6)	Inter-day (*n* = 3)	HQD	HQD
Paeoniflorin (1)	3.815–38.15	$y = 8 \times 10^7 \times + 13445$	$R^2 = 0.990$	863.58	4263.45	1.03	1.29	1.79	1.29
Liquiritin (2)	1.155–11.55	$y = 1 \times 10^9 \times + 38914$	$R^2 = 0.993$	67.46	325.50	1.12	2.02	2.98	1.02
Baicalin (3)	1.21–12.1	$y = 1 \times 10 \times + 3 \times 106$	$R^2 = 0.997$	360	3025	2.15	2.15	2.02	2.15
Oroxylin A-7-glucuronide (4)	2.125–34.0	$y = 3 \times 10^9 \times + 3 \times 106$	$R^2 = 0.991$	474.3	1517.85	2.79	2.09	2.14	2.32
Wogonoside (5)	4.25–34.0	$y = 3 \times 10^9 \times -7 \times 106$	$R^2 = 0.991$	2547.72	2421.84	2.23	1.29	1.23	1.23
Baicalein (6)	0.745–7.45	$y = 5 \times 10^9 \times -20357$	$R^2 = 0.999$	21.88	72.53	1.28	1.02	1.22	1.22
Wogonin(7)	0.113–2.25	$y = 1 \times 10^{10} \times +17890$	$R^2 = 0.999$	5.82	31.66	2.79	1.79	1.79	0.79
